# New insights into the cardiovascular risk of migraine and the role of white matter hyperintensities: is gold all that glitters?

**DOI:** 10.1186/1129-2377-14-9

**Published:** 2013-02-01

**Authors:** Claudio Tana, Emmanuele Tafuri, Marco Tana, Paolo Martelletti, Andrea Negro, Giannapia Affaitati, Alessandra Fabrizio, Raffaele Costantini, Andrea Mezzetti, Maria Adele Giamberardino

**Affiliations:** 1 Department of Medicine, “G. D’Annunzio” University of Chieti, and Center of Excellence on Aging, “G. D’Annunzio” University Foundation, Chieti, Italy; 2 Center of Excellence on Headache, “S.S. Annunziata” Hospital, Chieti, Italy; 3 Department of Clinical and Molecular Medicine, Regional Referral Headache Centre, “Sant’Andrea” Hospital, “Sapienza” University, Rome, Italy; 4 Institute of Surgical Pathology, “G. D’Annunzio” University, Chieti, Italy; 5 Private address: via Carlo de Tocco n. 3, Chieti, 66100, Italy

**Keywords:** Migraine, Cardiovascular events, Risk factors

## Abstract

The role of migraine as an independent risk factor for cardiovascular events has been debated for several years, while it is more established for ischemic stroke. Recently, new studies have examined the likelihood of migraine to determine cardiovascular events, supporting the hypothesis of a predominant role in patients with migraine with aura, the risk including both sexes. In the literature, multiple pathophysiological mechanisms are described to explain this association, and are here discussed. Furthermore, the emerging evidence that a higher headache frequency and long-term migraine may worsen the cardio-metabolic profile in migraineurs (e.g. with a higher Framingham risk score and risk of developing atherosclerosis, insulin resistance and metabolic syndrome) makes it increasingly necessary to reduce the number and severity of attacks, not only to alleviate the painful symptoms, but also to improve the prognosis in these patients.

## Introduction

Although mortality from cardiovascular disease (CVD) has considerably decreased in recent decades, mainly due to a better primary prevention (e.g. smoking cessation, diet and lifestyle changes), it still remains the leading cause of premature death in developed countries [1], constituting the underlying cause in 36.3% of cases in the United States [2].

Risk factors can be traditionally classified as non-modifiable (age and gender) and modifiable (e.g. history of hypertension, diabetes, smoking, lack of a regular diet and physical activity) [1-3].

In the case–control INTERHEART study, increased ApoB/ApoA1 ratio (OR 3.25 for top vs lowest quintile), smoking (2.87 for current vs never), history of diabetes (2.37), hypertension (1.91), abdominal obesity (1.12 for top vs lowest tertile and 1.62 for middle vs lowest tertile), psychosocial factors (2.67), lack of daily consumption of fruit and vegetables (0.70), regular alcohol consumption (0.91) and lack of regular physical activity (0.86) have been significantly associated with myocardial infarction in the United States (p < 0.0001 for all risk factors and p = 0.03 for alcohol) [3]. The role of migraine as a cardiovascular risk factor has been debated for years, while it seemed to be more established for ischemic stroke. Recent studies have therefore examined its likelihood to determine cardiovascular events, and these are discussed below. Furthermore, new data are available about the role of white matter hyperintensities (WMHs) in migraine patients [4].

## Review

### Migraine as an independent risk factor for vascular events: what’s new?

#### Recent evidence from the literature

Several studies have investigated the complex relationship between migraine and the risk of cardio- and cerebrovascular events, a possible link having already been hypothesized twenty years ago [5]. In a large prospective cohort study which enrolled, from the Women’s Health Study, 27,840 US migraineur women aged 45 years or older and free of angina and CVD at study entry, Kurth et al. reported 580 major CVD events during a mean follow-up of 10 years. Women who reported active migraine with aura (MA) had multivariable-adjusted hazard ratios of 2.33 (95% CI, 1.21-4.51; P = .01) for ischemic CVD death, 2.15 (95% confidence interval [CI], 1.58-2.92; P < .001) for major CVD, 2.08 (95% CI, 1.30-3.31; P = .002) for myocardial infarction, 1.91 (95% CI, 1.17-3.10; P = .01) for ischemic stroke, 1.74 (95% CI, 1.23-2.46; P = .002) for coronary revascularization, and 1.71 (95% CI, 1.16-2.53; P = .007) for angina, compared with women with no migraine history. Migraine without aura (MO) was not associated with an increased risk of any CVD event [6]. An increased risk of ischemic stroke had already been documented previously in different case control studies [7-9] and, subsequently, in a systematic review and meta-analysis of 14 observational studies published between 1966 and June 2004 (RR 2.16, 95% CI 1.89 to 2.48), which also showed an increased risk of these events both in patients with (RR 2.27, CI 1.61 to 3.19) and without aura (RR 1.83, CI 1.06 to 3.15), as well as in those taking oral contraceptives (RR 8.72, 5.05 to 15.05) [10].

Later, Schürks et al. confirmed a twofold increased hazard of ischaemic stroke in migraine patients, particularly women, affected with MA, aged < 45 years, smokers and with a history of the use of oral contraceptives. In contrast with previous data, the authors did not find an overall association between any migraine and myocardial infarction, death due to cardiovascular disease [11] or, in another work, mortality from all causes, CVD, or coronary heart disease (CHD). They documented, however, a moderate to high heterogeneity among included studies [12]. In men, migraine seems to have a major role in cardiovascular diseases, compared with nonmigraineurs; indeed, in a prospective cohort study of 20,084 men aged 40 to 84 years free of CVD at the start, who participated in the Physicians’ Health Study, Kurth et al. found, among 1449 men reporting migraine, 2236 major CVD events during a mean follow-up of 15.7 years, with multivariable-adjusted hazard ratios (95% confidence intervals) of 1.42 (1.15-1.77; P < .001) for myocardial infarction, 1.24 (1.06-1.46; P = .008) for major CVD, 1.12 (0.84-1.50; P = .43) for ischemic stroke, 1.15 (0.99-1.33; P = .068) for angina, 1.07 (0.80-1.43; P = .65) for ischemic cardiovascular death, and 1.05 (0.89-1.24; P = .54) for coronary revascularization, compared to patients without migraine. Therefore, this study highlights the important role of migraine as a cardiovascular risk factor in men, in particular for myocardial infarction. The main limit of this trial was the unavailability of data about aura; furthermore, male gender constitutes, per se, a risk factor for cardiovascular disease [13].

Recently, Gudmundsson et al. have again raised the hypothesis about migraine as an independent CV risk factor in both sexes, reporting, in a population based cohort study among 18,725 men and women born in the period 1907–35, an increased risk of all-cause mortality (adjusted - for sex and multivariables- hazard ratio 1.21, 95% CI 1.12 to 1.30), mortality from cardiovascular disease (1.27, 1.13 to 1.43), mortality from coronary heart disease (1.28, 1.11 to 1.49) and stroke (1.40, 1.10 to 1.78) in patients with migraine with aura compared with headache-free controls, while those with migraine without aura and non-migraine headache had no increased risk. The authors also showed that migraineur women had an increased risk of mortality from non-cardiovascular disease (1.19, 1.06 to 1.35) [14]. A post-hoc subgroup analysis of the Women’s Health Study, which randomized 100 mg aspirin on alternate days in primary prevention of CVD among 39,876 women aged ≥ 45 years, showed that aspirin reduced the risk of ischaemic stroke (RR, 0.76, 95% CI 0.63-0.93) but not of other CVD. Conversely, female patients with migraine with aura, who were assigned to aspirin, had an increased risk of myocardial infarction (RR 3.72, 95% CI 1.39-9.95); this was evident only for nonsmokers or those affected with hypertension (p < 0.01) [15]. Other authors have instead shown that migraine patients tend to have a significantly enhanced risk of venous thromboembolism (18.9% vs 7.6% in non-migraineurs, age/sex-adjusted p = 0.031) [16].

#### Common mechanisms for different diseases?

A putative link between migraine and the risk of cardiovascular events can be supported by the detection of higher concentrations of some serum markers in migraineurs compared to controls; pro-brain natriuretic peptide (pro-BNP) has been found to be elevated compared with healthy subjects (27.0 ± 28.0 versus 13.2 ± 8.6, p = 0.006), suggesting a preclinical cardiac involvement in these patients [17]. Serum from migraineurs, compared to controls, has also shown a condition of pro-inflammatory state: Uzar et al. have indeed documented the presence of higher levels of IL-1beta and IL-6 compared to controls (for IL-1beta; 5.73 ± 1.44 vs. 4.90 ± 1.40 pg/mL, respectively, p = 0.006; for IL-6; 3.1 ± 1.44 vs. 2.40 ± 0.22 pg/mL, respectively, p = 0.007) and lower values of IL-10 (3.38 ± 2.93 pg/mL) than healthy subjects (6.76 ± 1.48 pg/mL) (p = 0.007) [17]. Furthermore, in migraine patients, particularly those with aura, there was a significant reduction of the number and function of circulating endothelial progenitor cells [18] (EPCs, which appear to be involved in repair and angiogenesis of ischemic tissues [19]), compared to controls and patients with tension type headache (8.6 ± 10.1 in MA versus 20.4 ± 22.2 in MO, p = 0.001; 47.8 ± 24.3 in TTH versus MO, p < 0.001). This finding, indicating a reduced migratory capacity and increased cellular senescence in migraineurs, suggested that EPCs are another underlying link between migraine and cardiovascular risk [18]. The presence of altered endothelial function in migraineurs has recently been confirmed by Rodríguez-Osorio et al., who analyzed flow-mediated dilation (FMD) in the dominant brachial artery, calcitonin gene-related peptide (CGRP), and vascular endothelial growth factor (VEGF) levels by ELISA, nitric oxide stable metabolites (NOx) by high-performance liquid chromatography, and EPCs in peripheral blood samples of 47 patients with a diagnosis of episodic migraine performed according to the International Headache Society (IHS) 2004 criteria, and of 23 control subjects, obtained during interictal periods and migraine attacks. Compared to controls, migraineurs showed reduced levels of EPCs (9.4 ± 5.0 vs 17.9 ± 6.0; p < 0.0001), higher values of NOx (1225.2 ± 466.1 vs 671.9 ± 358.6 μM), VEGF (473.4 ± 398.7 vs 72.6 ± 56.6 pg/mL), and CGRP (164.2 ± 139.1 vs 37.1 ± 38.5 pg/mL) (p < 0.05); higher levels of these markers were reported during attacks. An interesting result was that EPC counts decreased as migraine progressed in time [20]. An adverse cardiovascular profile in patients with migraine may also be explained by the detection of a condition of oxidative stress, in terms of both an increase in oxidising substances and antioxidant mechanisms [21]. Malondialdehyde (MDA) levels and erythrocyte superoxide dismutase (SOD) activity were, indeed, significantly higher in migraineurs than in controls, as shown by Tuncel et al. The SOD activity was significantly higher in the MA subgroup as compared to MO [22]. The levels of 4-hydroxy-2-nonenal (HNE), increased in female patients compared to controls (OR for migraine of 4.55), were significantly correlated with the nitric oxide pathway, insulin- and lipid-metabolism [23]. Furthermore, Gupta et al. assessed the oxidative stress in migraineurs and TTH subjects by collecting a venous blood sample from the antecubital vein at least 3 days after the last headache attack. They found higher values of malondialdehyde (MDA) and ferric reducing activity of plasma (FRAP) in migraine patients than in the subjects with TTH and controls (P < 0.001). Compared to MO, FRAP levels were significantly higher in patients with mixed migraine (MA and MO, P = 0.01) [24]. Migraineurs have also been shown to present higher concentrations of nitric oxide (NO, 35.6 ± 7.7, 31.0 ± 6.2 μmol/L, respectively, p = 0.005) and asymmetric dimethylarginine (ADMA, 0.409 ± 0.028, 0.381 ± 0.044 μmol/L, respectively, p = 0.001) in comparison with controls. During the crises, NO and ADMA concentrations were significantly higher in migraine patients compared to healthy subjects (p = 0.015 and p = 0.014, respectively) [25].

**Figure 1:**
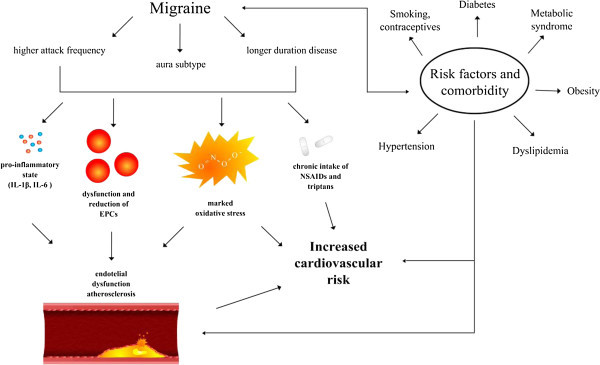
**Putative mechanisms which may increase the risk of vascular events in patients with migraine.** EPCs Endothelial Progenitor Cells; NSAIDs Non-steroidal anti-inflammatory drugs.

As far as NO and ADMA are concerned, migraine patients compared to controls showed higher levels of platelet peroxynitrite (ONOO-), a reactive oxidant derived from superoxide anion and NO (P < 0.001); it has been suggested that this could be related to changes in fluidity and activity of platelet membrane [26]. Lastly, it is interesting to note that N-acetyl-aspartate (NAA), a serum biomarker of neuronal integrity synthesized in neural mitochondria [27], has recently been found to be significantly decreased in migraineurs (0065 ± 0019 mol/L, with lower levels in those with aura), as compared with both tension-type headache patients (0078 ± 0016 mol/L, p = 0.002) and controls (0085 ± 0013 mol/L, p <0.0001) [28]. Reduced levels of NAA could be associated with an increased risk of stroke and migraine progression [27]. Figure 1 shows the mechanisms which may increase the risk of vascular events in migraine patients.

### Cardiovascular risk profile in patients with migraine and interactions between risk factors

#### The Framingham risk score

In all asymptomatic adults without a clinical history of CHD, the risk of cardiovascular events should be assessed by global risk scores, such as the Framingham Risk Score in the American population, which combines multiple traditional cardiovascular risk factors (such as age, sex, total cholesterol, HDL cholesterol, smoking, systolic blood pressure, antihypertensive medications) into a single quantitative estimate of risk which can be used to target preventive interventions [29,30]. Several trials have evaluated the Framingham risk score in the population of migraineurs; the recent cross-sectional population-based HUNT study enrolled 48,713 subjects (age ≥ 20 years) and in 44,098 (90.5%) of these assessed parameters such as blood pressure (BP), body mass index (BMI), serum total and high-density lipoprotein cholesterol to calculate the Framingham 10-year risk score for coronary death and myocardial infarction. The authors found an unfavourable cardiovascular risk profile, established by an elevated Framingham risk score, both in migraine with (OR 1.54, 95% CI 1.21-1.95) and without aura (OR 1.17, 95% CI 1.04-1.32) and in non-migrainous headache (OR 1.17, 95% CI 1.10-1.26). As shown above, the risk was more pronounced in patients with migraine with aura, confirming its major link with CV diseases. A very interesting result was that the Framingham risk score consistently increased with a higher headache frequency. For non-migrainous headache and migraine without aura, the increased risk was accounted for by lifestyle factors such as low physical activity, smoking and a high BMI; conversely, these factors did not completely explain the elevated risk in the patients with migraine with aura, suggesting other mechanisms underlying the elevated risk in these than in other headache types [31].

#### Metabolic syndrome and its components

The complexity of the relationship between migraine and the risk of cardio and cerebrovascular diseases may be partly explained by the detection of a higher prevalence of multiple risk factors in these patients than controls; this may suggest an interaction between them, enhancing the CV risk profile in migraineurs. These patients have indeed demonstrated a higher prevalence of metabolic syndrome than the general population [32,33], with a correlation with age, gender, number of triggers, years of headache and duration of migraine attacks [33].

From the Genetic Epidemiology of Migraine (GEM) population-based study conducted in the Netherlands, 620 patients with migraine were identified among 5,755 enrolled participants. Compared to controls, they were more likely to be smokers (OR = 1.43 [1.1 to 1.8]) and have a parental history of early myocardial infarction, and they were less likely to be alcohol consumers (OR = 0.58 [0.5 to 0.7]). Furthermore, migraine with aura patients were more likely to have elevated blood pressure (systolic BP > 140 mm Hg or diastolic BP > 90 mm Hg [OR = 1.76 (1.04 to 3.0)]), a worse cholesterol profile (TC > or = 240 mg/dL [OR = 1.43 (0.97 to 2.1)], TC:HDL ratio > 5.0 [OR = 1.64 (1.1 to 2.4)]), and an earlier onset of CHD or stroke (OR = 3.96 [1.1 to 14.3]), with doubled odds of having an elevated Framingham risk, resulting in a higher CV risk profile than patients without migraine [34].

#### Lipidic profile

According to these data, the recent population-based study by Bigal et al. found that migraineurs, compared to controls, were more likely to have a diagnosis of hypercholesterolemia (32.7% vs 25.6%, OR 1.4, 95% CI 1.3-1.5), but also diabetes (12.6% vs 9.4%, OR 1.4, 95% CI 1.2-1.6) and hypertension (33.1% vs 27.5%, OR 1.4, 95% CI 1.3-1.6). The risk was highest in patients with migraine with aura, but remained increased in those without aura, with significant higher Framingham scores in both than in controls. After adjustments for CVD risk factors, gender, age, disability and treatment, migraine remained significantly associated with myocardial infarction (OR 2.2, 95% CI 1.7-2.8), stroke (OR 1.5, 95% CI 1.2-2.1), and claudication (OR 2.69, 95% CI 1.98-3.23) [35].

A significant elevation of total cholesterol and triglycerides was also found among migraine with aura patients enrolled in the cross-sectional Epidemiology of Vascular Ageing Study: the OR (95% confidence interval) was indeed 4.67 (0.99-21.97) for the 2nd tertile and 5.97 (1.29-27.61) for the 3rd tertile of total cholesterol and OR of 4.42 (1.32-14.77) for the 3rd tertile of triglycerides. A significant association between increased biomarker levels and any other headache group was not found [36].

#### Obesity

Obesity has been found to be another important cardiovascular risk factor associated with migraine, especially with a new onset chronic daily headache (CDH), higher frequency and intensity of migraine crises [37-39]. With respect to BMI, Bigal et al. have indeed shown an increase in the proportion of subjects with severe headache pain and a doubling of cases in the morbidly obese group (BMI > or = 35 kg/m(2)) compared to the normally weighted group (BMI 18.5 to 24.9 kg/m(2); OR = 1.9). A greater BMI was, indeed, directly correlated with an elevated number of headache crises [4.4% of cases with 10 to 15 headache days per month in the normally weighted group, 5.8% in the overweight (OR = 1.3), 13.6% in the obese (OR = 2.9), and 20.7% in the morbidly obese group (OR = 5.7)] [38].

Furthermore, for a BMI > or = 35 kg/m(2), an OR of daily migraine of 3.11 (1.12, 8.67) has been shown in women and, among those with an active form, a higher risk was present of phonophobia and photophobia but not of migraine with aura, which was instead decreased [39].

#### Hyperinsulinemia and glucose tolerance

In addition to the risk factors discussed above, there is growing evidence that alterations of the glucose metabolism may be related to migraine and involved in its pathogenesis. In a study which enrolled 84 patients with migraine (73 F, 11 M), 25 patients with non-migraine headache (20 F, 5 M), and 26 healthy subjects (24 F, 2 M), some authors showed the presence of higher levels of insulin in migraine than in other headaches (P < .0001) and controls (P < .0001), and higher levels of blood glucose in headache patients, with a greater difference between migraine and healthy subjects (P < .0001) than between other headaches and controls (P < .05). In this study, therefore, unlike glucose levels, hyperinsulinemia appeared to be specific for migraine patients [40]. Moreover, a higher prevalence of insulin resistance has been found [33,41], as well as a correlation of this with the duration of migraine attacks [33]. Similar data were obtained by Gruber et al., who enrolled one hundred and twenty non-obese subjects, including 48 migraineurs and 72 healthy volunteers; they found high levels of insulin in migraine patients and also showed a link between migraine-related hyperinsulinemia and increased NO stress [42]. Correlated with high levels of insulin, leptin and GLP-2 levels were also found to be increased in 84 non-obese female patients, revealing ORs of 3.79 and 4.26 for migraine, respectively, when comparing the lowest with the highest quartile of the test variable in the complete study cohort by logistic regression analysis [43].

### Migraine and subclinical organ damage

#### Microvascular abnormalities

In view of the increased susceptibility of patients with migraine to vascular events, it would be useful to investigate the presence of subclinical organ damage, which may further increase the cardiovascular risk in migraineurs. Carotid intima-media thickness (CIMT) is increasingly used as a surrogate marker for atherosclerosis [44], and has proved to be higher in patients with migraine than in healthy controls. After matching for gender, age, BMI, blood pressure, and cholesterol, the real-time gray-scale ultrasound examination of the left common carotid arteries (CCA) of 30 patients with a diagnosis of episodic migraine, carried out according to the IHS criteria, and of 60 healthy controls aged between 20 and 40 years, showed the presence of significantly higher IMT values in migraineurs, with a mean CCA-IMT of 0.493 ± 0.074 mm in migraine patients and of 0.409 ± 0.053 mm in controls (P < 0.001). The authors of the study concluded that the risk of arteriopathy increased with the repeating of the migraine attacks, supporting the role of the severity of attacks (in terms of number and duration) in the development of atherosclerosis [45]. In migraineurs, CIMT was also correlated with systolic blood pressure (SBP, p < .01), total cholesterol (P < .01), triglycerides (P < .001), glucose (P < .001), insulin (P < .01) and BMI (P < .05). The artery flow-mediated dilatation (FMD) in response to hyperemia was instead inversely correlated with these parameters, in particular SBP (P < .001), DBP (P < .01), glucose (P < .001) and insulin levels (P < .01). Plasma endothelin (ET)-1, a marker of endothelial injury and atherosclerosis released by vascular smooth muscle cells, was increased in these patients and correlated with the duration of illness, IMT, FMD% and other clinical-laboratory parameters (SBP, DBP, insulin and glucose levels) [46].

A sub-study of the Atherosclerosis Risk in Communities Study has also found that patients with headache, compared to those without a history of it, were more likely to present retinopathy (OR = 1.49, 95% CI = 1.05 to 2.12 for migraine without aura; OR = 1.38, 95% CI = 0.96 to 1.99 for migraine/other headaches with aura; and OR = 1.28, 95% CI = 0.99 to 1.65 for other headaches), with a stronger association in the subset of patients without a history of hypertension or diabetes (OR = 1.79, 95% CI = 1.09 to 2.95 for migraine/other headaches with aura; and OR = 1.74, 95% CI = 1.11 to 2.71 for migraine without aura). The most frequently reported sign of retinopathy consisted of a reduction of mean arteriolar and venular calibers with respect to controls [47].

### The role of white matter hyperintensities

#### Definition, pathophysiology and clinical relevance in headache-free patients

White matter hyperintensities can be defined as small, non-specific, multiple lesions of the brain, usually located in the periventricular or deep white matter. They do not create mass effect on the surrounding parenchyma and mainly appear as hyperintense on T2-weighted and FLAIR images [4]. Microscopically, they are characterized by the presence of local loss of myelin, axons and oligodendroglial cells and by a mild reactive astrocytic gliosis [48]. Many theories have been proposed to explain their formation, the most accepted being that white matter hyperintensities are the result of an incomplete process of ischemia due to arteriolosclerosis of small vessels in the brain. They are indeed mainly related to the aging process and multiple vascular risk factors, such as hypertension, elevated BMI, hypercholesterolemia and diabetes [48-52]. Other proposed theories are blood–brain barrier dysfunction due to small vessel alterations, and consequent chronic diffusion of macromolecules and fluids in the white matter; oxidative stress, endothelial dysfunction and dysfunction of mechanisms of vascular regulation [48]. A large amount of data have documented the clinical relevance of these lesions in non-headache people; WMHs have indeed been associated with cognitive impairment (both in terms of global cognition, executive function, mental and processing speed), long-term cognitive decline, gait disturbance and falls, stroke, death, depression and urinary incontinence [48,53-60].

#### WMHs in migraine patients: recent evidence

Several studies have documented a higher prevalence of these lesions in migraineurs (both with and without aura) than in control subjects, after adjustment for risk factors [61-63].

In a study which randomly selected 134 patients with MO, 161 with MA, and 140 controls from the GEM study, after adjustment for migraine type, attack frequency, age at onset and treatment status, Kruit et al. found the presence of WMHs in 1 of 140 controls (0.7%) and in 13 of 295 patients with migraine (4.4%; *P* = 0.04), slightly more in MA (8 of 161; 5.0%) than in MO (5 of 134; 3.7%) [64]. According to previous data [63], they found an increased prevalence of periventricular and subcortical white matter lesions (PVWMLs and DWMLs, respectively), but not of cerebellar infarcts, unlike their earlier study. In the majority of cases (86%), the lesions were located in the pons (pontine hyperintense lesions [PHLs]) [64]. The recent Cerebral Abnormalities in Migraine, an Epidemiological Risk Analysis (CAMERA) study, which randomly selected 295 patients with migraine and 140 controls from a previously diagnosed population-based sample (n = 6039), showed a higher prevalence of subclinical infarcts in the posterior circulation, most pronounced for patients who were migraineurs with aura [OR 13.7; 95% CI 1.7, 112], and of brainstem hyperintense lesions (4.4% vs. 0.7%, P = 0.04). Women with migraine had an independent increased risk of WMLs (OR 2.1; 95% CI 1.0, 4.1). The authors found other interesting results, such as a higher lifetime prevalence of syncope and orthostatic insufficiency in migraine patients and, in those aged < 50 years, the presence of increased iron accumulations in red nucleus (P = 0.03) and basal ganglia such as globus pallidus (P = 0.03) and putamen (P = 0.02) [65].

#### Pathophysiology and clinical relevance of WMHs in migraine patients

The reason for the higher prevalence of WMHs in migraineurs is still debated: the subclinical ischemia theory is the most attractive. The higher prevalence of these phenomena in patients with aura has been firstly attributed to the occurrence of repeated oligoemia, that may lead to a local hypoperfusion and to minor brain injury [4], and, more recently, to changes in the permeability of the blood– brain barrier by activation of matrix metalloproteinases (MMPs), a family of neutral metalloproteases, due to the oxygen free radicals, nitric oxide and proteases highly released during the spreading depression, a self-propagating wave of neuronal and glial depolarization which generates a transient loss of membrane ionic gradients [66].

This hypothesis, however, does not explain the development of such lesions in patients with migraine without aura, who present them similarly to migraineurs with aura, although to a lesser degree [65]. In MO patients, a higher attack frequency and longer disease duration have been called upon to explain the development of WMHs [4,65,67,68]. These data are concordant with our recent findings, which demonstrated a similar prevalence of WMHs both in migraine without aura and tension-type headache, and suggested a major link between the frequency (rather than the intensity) of crises and MRI positivity [69], and with those of Kurth et al., which associated any history of severe headache with the increasing volume of white matter hyperintensities [70]. The above-mentioned effects of risk factors, comorbidity and other mechanisms (e.g. endothelial dysfunction, assumption of triptans with vasoconstrictor activity) may also contribute to the development of these lesions [17-26,68].

The recent finding that these injuries, like those of headache-free patients, may progress over time raises some doubts about their benign nature: among 26 subjects with WMHs, 8 patients had indeed new lesions after a follow-up of a mean duration of 33.2 months [71]. However, in contrast to previously published data [72], there is at the moment no evidence that migraine, other primary headaches or migraine-related medication use are risk factors for cognitive dysfunction or cognitive deterioration over time [70,73]. Furthermore, Serafini et al. found that patients with PWMHs reported fewer depressive symptoms than patients without PWMHs, and those with WMH lesions reported less severity of their depression state [74].

## Conclusions: implications in clinical practice

The growing evidence of a greater risk of ischemic stroke in patients with migraine, particularly in the case of aura, female sex, smoking habits, age < 45 years and a history of oral contraceptive assumption [11,14], the recent finding of a cerebral protective effect of aspirin (in terms of a reduction of the risk of ischaemic stroke, in the dose of 100 mg on alternate days) [15], and of the risk, although already debated [6,11-13], of myocardial infarction in migraine with aura, raise some doubts about the complete benignity of migraine [75]. A migraineur subject, even with a low absolute CV risk (female, age under 45 years) may, in fact, be at a higher relative risk due to the presence of other factors, such as aura component, smoking and the use of oral contraceptives. It is therefore important to act on these factors in order to reduce the vascular hazard. In addition, because the higher headache frequency and long-term migraine may result in a worse cardiometabolic profile, in terms of a higher Framingham risk score [26], greater risk of development of atherosclerosis [45], insulin resistance [30,33] and metabolic syndrome [33], it may be useful to reduce the number and severity of attacks. This is not only to alleviate the painful symptoms, but also to improve the prognosis in these patients, also considering that a higher frequency of attacks may be related to a history of chronic intake of non-steroidal anti-inflammatory drugs (NSAIDs) and triptans, both well known to be associated with a greater likelihood of developing cardiovascular events. At present, however, there is a lack of evidence that the presence of white matter hyperintensities, although possibly correlating positively with a higher attack frequency and duration of the disease [69,70], can deteriorate the cognitive function and worsen -per se- the risk of cardiovascular events in migraine patients [70,73]. If it proves true that they are indicative of CV risk in migraine, these WMHs, by revealing the presence of subclinical organ damage, could be a useful marker in migraineurs to evaluate the increased hazard for CV events as well as the presence of carotid intima-media thickening and atherosclerosis in the general population. New longitudinal studies are needed to draw definite conclusions in the future.

## Competing interests

The authors declare that they have no competing interests.

## Authors’ contribution

All authors of this manuscript (CT, ET, MT, PM, AN, GA, AF, RC, AM, MAG) have made substantial contributions to conception and design of the review, have been involved in drafting the manuscript and revising it critically for important intellectual content and have given final approval of the version to be published.
